# Ending tuberculosis in Gulf Cooperation Council countries: an overview of the WHO End TB Strategy 2025 milestones

**DOI:** 10.1016/j.ijregi.2025.100681

**Published:** 2025-06-06

**Authors:** Salah Al Awaidy, Faryal Khamis, Sami Al Mujeini, Jameela Al Salman, Jaffar A. Al-Tawfiq

**Affiliations:** 1 Freelance Public Health Consultant, Muscat, Seeb, Oman; 2 Adult Infectious Diseases, Department of Medicine, Royal Hospital, Ministry of Health, Muscat, Oman; 3 Department of Communicable Diseases and Control, General Directorate of Health Services, Ministry of Health, South Al Batinah Governorate, Oman; 4 Medical Department, King Hamed American Mission Hospital, A’Ali, Manama, Bahrain; 5 Arabian Gulf University, Manama, Bahrain; 6 Infectious Disease Unit, Specialty Internal Medicine, Johns Hopkins Aramco Healthcare, Dhahran, Saudi Arabia; 7 Division of Infectious Diseases, Department of Medicine, Indiana University School of Medicine, Indianapolis, USA; 8 Division of Infectious Diseases, Department of Medicine Johns Hopkins University, Baltimore, USA

**Keywords:** TB, TB elimination, End of TB, WHO End TB Strategy, GCC countries

## Abstract

**Objectives:**

Tuberculosis (TB) continues to pose a major global public health challenge. Although the Gulf Cooperation Council (GCC) countries have lower incidence rates of TB (<10 cases per million population), they are still striving to maintain and surpass benchmarks. We describe TB status to the 2025 milestones of the World Health Organization (WHO) End TB Strategy.

**Methods:**

A retrospective study conducted using WHO data published from 2015 and between 2020 and 2023 highlights trends in TB incidence, mortality, treatment success, and drug resistance across GCC countries.

**Results:**

Kuwait had the most significant drop in TB infections since 2015, with a reduction of 57%, followed by Qatar at 40% and Saudi Arabia at 31%. Oman, however, recorded a 23% increase. In terms of TB-related deaths, Saudi Arabia achieved a 32% decrease, whereas Qatar, Kuwait, and the United Arab Emirates experienced increases of 14%, 39%, and 22% respectively. Treatment success rates were highest in Qatar (100%), Oman (90%), and Saudi Arabia (87%), whereas Kuwait (44%) and Bahrain (77%) had the lowest rates. Oman reported the highest reduction in multidrug-resistant TB cases at (60%), followed by Saudi Arabia (50%), Kuwait (31%), and Bahrain (50%). Qatar had the highest increase (122%) and United Arab Emirates had no change, reflecting varying degrees of success in controlling drug-resistant TB. HIV-TB co-infection rates was highest in United Arab Emirates (5.4%) and Bahrain (5.1%), followed by Saudi Arabia (2.3%), Oman (1.4%), Qatar (0.6%), and Kuwait (0.5%). Notably, the GCC countries reported zero TB households, with no catastrophic overall expenses since 2020.

**Conclusions:**

Although the GCC countries have made significant strides in TB control, their efforts toward the targets have been inconsistent, with notable differences in TB-related deaths, infection rates, treatment success, and multidrug-resistant TB rates. Strengthening regional collaboration, implementing targeted interventions, and integrating services are essential to meet the WHO’s 2025 and 2035 goals.

## Highlights

• Tuberculosis (TB) continues to pose a major global public health challenge.
• The Gulf Cooperation Council countries have low-incidence TB rates.
• The Gulf Cooperation Council countries have made significant strides in enhancing the TB program; however, their efforts have been inconsistent.
• Collaborative approach and targeted activities are essential to align strategy goals.

## Introduction

Tuberculosis (TB) remains a global health threat, as indicated by the World Health Organization (WHO)’s Global Tuberculosis Report 2023. Approximately 10.8 million new cases were reported worldwide in 2023, marking an increase from previous years. The number of cases climbed from 10.1 million in 2020 to 10.4 million in 2021 and 10.7 million in 2022. The upward trend per 100,000 individuals from 129 to 134 represents a 4.6% rise in the global TB incidence rate between 2020 and 2023. This troubling rise comes after a decade of annual reductions of 2% in TB cases between 2010 and 2020 [1].

Although, regionally, the TB incidence rates exhibited varying trends in 2023, with an upward trend in the Americas and Western Pacific regions and a slight decline in the Eastern Mediterranean and Southeast Asian regions, proceeded by 2 years of increase. Furthermore, TB has reemerged as a major public health concern, likely becoming the leading cause of death from a single infectious agent in 2023, surpassing COVID-19. TB-related deaths are approximately double those caused by HIV/AIDS, underscoring the severity of the TB epidemic [1].

In 2014 and 2015, all WHO and United Nations Member States committed to end the TB pandemic by adopting the WHO End TB Strategy and the United Nations Sustainable Development Goals. The strategies set milestones for 2020 and 2025, as well as objectives for 2030 and 2035, to significantly reduce TB incidence rates, fatalities, and expenses for individuals and their families [1].

The WHO End TB Strategy outlines specific milestones for achieving these goals. By 2030, the strategy aims for a 75% reduction in the absolute number of TB deaths compared with the 2020 baseline, a 50% reduction in the TB incidence rate, and zero TB-affected households facing catastrophic costs. These objectives highlight the need for sustained efforts to combat TB worldwide, emphasizing the importance of effective strategies and international collaboration to address this persistent health threat [1].

The National Plan for Accelerating Efforts to End TB 2021-2025, derived from the National Strategic Plan for Ending TB, is a comprehensive strategy developed through a country-led, person-centered approach with WHO assistance. This plan outlines seven key objectives to combat TB effectively by 2025. These objectives include detecting 90% of incident cases, ensuring successful treatment for over 90% of enrolled patients, providing multidrug-resistant (MDR) TB diagnostic services to all presumptive cases and treating 90% of diagnosed patients with MDR-TB, implementing timely and accurate reporting from all centers, ensuring 100% availability of quality TB services delivered by qualified personnel, scaling up patient support systems to reduce catastrophic costs for all patients with TB, including those with drug-susceptible TB, and supporting operational research to foster innovation. Together, these objectives form a holistic approach to ending the TB epidemic by improving detection, treatment, management, data accuracy, service quality, patient support, and research efforts [2].

Although the global TB incidence has shown variable trends, the Gulf Cooperation Council (GCC) countries exhibit unique epidemiologic features influenced by demographic shifts, health care infrastructure, and socioeconomic factors.

The GCC is a regional intergovernmental union that encompasses Bahrain, Kuwait, Oman, Qatar, Saudi Arabia, and the United Arab Emirates (UAE). The GCC countries are low–TB incidence countries, where the rate of all forms of TB notification has consistently decreased to less than 100 cases per million population [1,3,4]. This overview highlights the progress made within the GCC countries in combating TB, with a particular focus on pulmonary TB. It explores the challenges faced by national TB programs (NTPs) and emphasizes key lessons learned. Furthermore, the overview presents recommendations for future actions necessary to achieve the WHO TB-related targets. By assessing the current situation, identifying obstacles, and proposing solutions, we aim to contribute to the ongoing efforts to control and untimely eliminate TB in the GCC region, in alignment with global WHO strategies and targets. We also specifically address the TB therapy success rates, rates of MDR-TB, and potential gaps and obstacles.

## Methods

This overview is based on data obtained from the WHO Global Tuberculosis Reports. We collected and compared TB incidence rates, mortality rates, and treatment outcomes in the GCC countries for two periods: 2015 and 2020-2023. The analysis is descriptive in nature, relying primarily on WHO data sets, with minimal statistical analysis conducted beyond direct comparison of reported figures. The End TB Strategy uses the year 2015 as the baseline year for comparison. The data set has been sourced from the WHO global TB reports and based on the WHO End TB Strategy’s indicators. The indicators include percentage reduction in number of TB deaths by 2020-2023 compared with 2015, percentage reduction by 2020-2023 in TB incidence rate compared with 2015, and percentage reduction of TB-affected families facing catastrophic costs due to TB by 2020-2023 [1]. Furthermore, between 2020 and 2024, we gathered and analyzed data on strategies for optimizing TB treatment, focusing on improving patient care through supportive services for vulnerable populations, such as migrants and people living with HIV (PLHIV).

## Results

### Tuberculosis incidence rate

**Figure 1:**
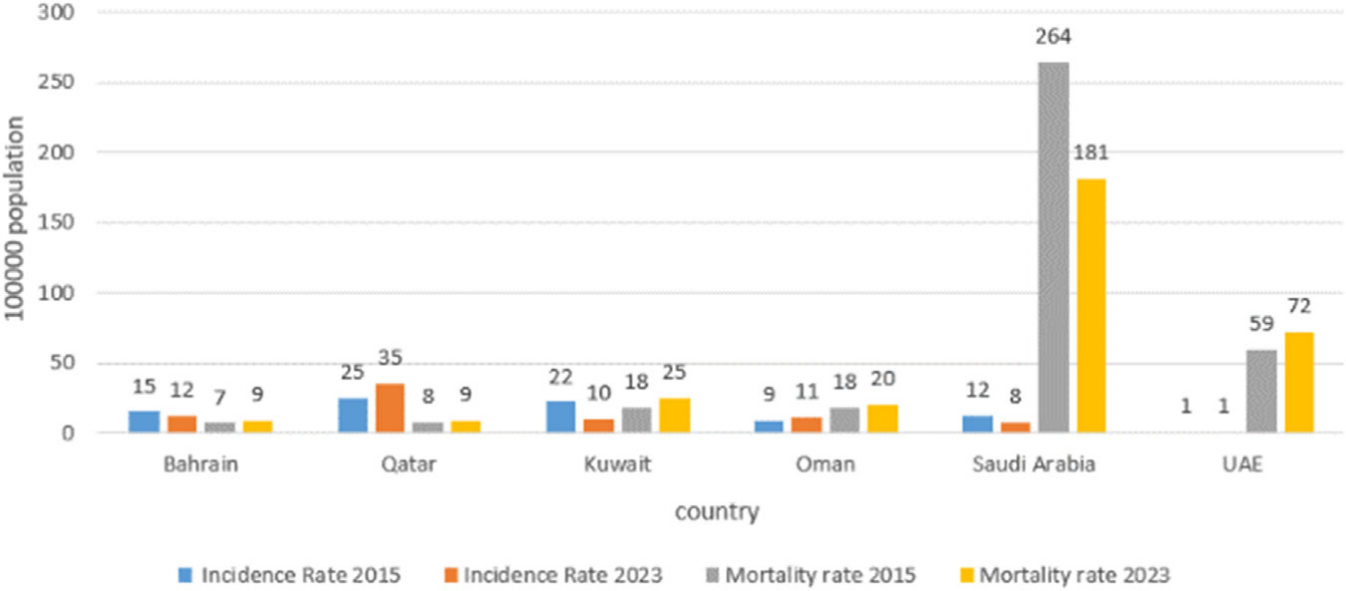
Incidence and mortality rate (per 100,000 population), Gulf Corporation Council countries, 2015-and 2023.

During the study period between 2020 and 2023, Bahrain and Kuwait experienced a decline in estimated TB incidence rate, demonstrating a success in efforts to combat the disease. Bahrain decreased the TB rate from 15 to 12 per 100,000 population, whereas Kuwait reduced the incidence rate from 18 to 10 cases per 100,000 population. Unfortunately, Qatar reported a persistently relatively high TB rate of 35 cases per 100,000 population, and Oman had an increase from 8 to 11 cases per 100,000 population. In contrast, the UAE had the lowest TB incidence rate in the region, reporting one case per 100,000 population (Table 1) (Figure 1).

**Table 1:** Tuberculosis incidence rate per 100,000 population, Gulf Corporation Council countries, 2020-2023.

Country	Tuberculosis incidence rate per 100,000 population			
	2020	2021	2022	2023
**Bahrain**	15	15	14	12
**Kuwait**	18	19	11	10
**Qatar**	35	40	35	35
**Oman**	8	6	9	11
**Saudi Arabia**	9	9	9	8
**UAE**	1	1	1	1

Source: [1].

### World Health Organization 2025 milestones of the End TB Strategy

Progress toward the 2025 goals of the End TB Strategy has been inconsistent across the GCC countries. Kuwait led the way with a 57% reduction in TB cases since 2015, followed by Qatar with a 40% reduction and Saudi Arabia with a 31% reduction. Unfortunately, there was a 23% increase in the reported cases from Oman (Table 2).

**Table 2:** The World Health Organization 2025 milestones of the End TB Strategy, 2015-2023.

Country	Change in TB incidence rate (estimated number of cases for 2015 and 2023)	Change in total TB mortality (estimated number of cases for 2015 and 2023)	Treatment success rate (2022)	Percentage of multidrug-resistant/rifampicin-resistant-TB (estimated number of cases for 2015 and 2023)	HIV-TB co-infection rate
**Bahrain**	20% ↓ (15-12)	130% ↑ (7-9)	77%	20% ↓ (5-4)	5.1%
**Qatar**	40% ↑ (25-35)	14% ↑ (8-9)	100%	122% ↑ (9-20)	0.6%
**Kuwait**	57% ↓ (22-10)	39% ↑ (18-25)	44%	31% ↓ (16-11)	0.5%
**Oman**	23% ↑ (9-11)	11% ↑ (18-20)	90%	60% ↓ (5-8)	1.4%
**Saudi Arabia**	31% ↓ (12-8)	32% ↓ (264-181)	87%	50% ↓ (90-45)	2.3%
**UAE**	Same (1 case per 100,000) (1-1)	22% ↑ (59-72)	N/A	same (1-1)	5.4%

Source: [1].

TB, tuberculosis.

https://worldhealthorg.shinyapps.io/tb_profiles/?_inputs_&tab=%22charts%22&lan=%22EN%22&iso2=%22QA%22&entity_type=%22country%22.

Saudi Arabia reported a 32% reduction in mortality rates due to TB. In contrast, mortality rates increased in Qatar by 14%, in Kuwait by 39%, and in the UAE by 22%. The countries with the highest treatment success rates were Qatar (100%), followed by Oman (90%) and Saudi Arabia (87%). On the other hand, Bahrain (77%) and Kuwait (44%) had lower success rates (Table 2). Oman had the highest reduction in MDR and rifampicin-resistant TB (RR-TB) (60%), followed by Saudi Arabia (50%), Kuwait (31%), and Bahrain (50%). Qatar had the highest increase (122%) and UAE no change, reflecting varying degrees of success in controlling drug-resistant TB. The UAE (5.4%) and Bahrain (5.1%) reported the highest HIV-TB co-infection rates, followed by Saudi Arabia (2.3%), Oman (1.4%), Qatar (0.6%), and Kuwait (0.5%) (Table 2).

The GCC countries have achieved a zero percentage of TB-affected households, with zero catastrophic total costs in 2020, due to the provision of free TB services to all populations that included free-of-charge diagnosis and treatment for patients and their households (Table 2).

### Management of in latent tuberculosis infection the Gulf Corporation Council countries

Management of latent TB infection (LTBI) in the GCC countries presents challenges and opportunities as the region strives toward TB elimination [5]. Despite the potential for achieving TB elimination, several obstacles must be addressed to meet established targets. The overall prevalence of LTBI in the Middle East and North Africa (MENA) region is notably high, with an overall prevalence rate at 41.78%, and considerable variability in the prevalence rate from different studies in the region, ranging from 0.44% to 88.15% [6].

## Discussion

A significant achievement across all GCC countries is the zero percentage of TB-affected households facing catastrophic costs due to the disease, attributed to the nominally of free TB services in the region [4]. This accomplishment aligns with the End TB Strategy’s goal of eliminating financial burdens on patients with TB and their families. The WHO defines catastrophic costs as total costs (including direct medical costs, direct non-medical costs, and indirect costs) exceeding 20% of the household’s annual income [7,8]. The provision of free TB services in GCC countries typically includes screening, diagnosis, follow-up care and monitoring, and medications [4]. This comprehensive approach not only ensures proper medical care but also addresses the socioeconomic aspects of TB, which can be a significant barrier to treatment adherence and successful outcomes.

Three of the GCC countries have shown important progress in combating TB between 2020 and 2023, with a decline in the incidence rates. This overall positive trend reflects the effectiveness of TB control strategies, aligning with global efforts to combat TB. The UAE stands out with an exceptionally low and consistent rate of one case per 100,000 population throughout the study period. This remarkable achievement sets a benchmark for the region and suggests highly effective TB control measures, including rigorous screening of immigrants, excellent health care infrastructure, and possibly successful latent TB treatment programs. Bahrain has demonstrated notable improvement, reducing its TB incidence rate from 15 to 12 cases per 100,000 population, representing a 20% decrease. Kuwait’s progress stands out as particularly impressive, with a substantial decrease from 18 to 10 cases per 100,000 population, marking a 44% reduction. This dramatic improvement likely indicates strong political commitment, enhanced health care infrastructure, and effective public health interventions. In Saudi Arabia, the decline in TB incidence can be attributed to the implementation of vaccination programs and improved access to health services. Based on the last global burden of disease, the world did not meet the first interim milestones of the WHO End TB Strategy in 2020 [9]. The data for Saudi Arabia showed a change of ≥−15% and <−10% for all-form TB incidence rate from 2015 to 2020 and of −45% for MDR-TB prevalence over the same period. Saudi Arabia’s TB incidence rate fell from 10.55 per 100,000 in 2015 to 8.76 per 100,000 in 2019 (pre-pandemic), marking a −17% decline. This surpassed the WHO’s annual 4-5% reduction target for the End TB Strategy. MDR-TB cases dropped sharply from 4.4% to 2.4% of total TB cases between 2015 and 2019 (*P* = 0.008) [10,11]. It is important to note that different regions of the same country may also have different levels of drug resistance. In a systematic review of 22 studies from Saudi Arabia published between 1979 and 2013, a significant heterogeneity was observed in the data. The prevalence rates of resistance to anti-tuberculous agents were as follows: isoniazid (INH) at 10.13%, rifampicin at 5.41%, ethambutol at 1.29%, and streptomycin at 6.5%. The prevalence of MDR-TB was recorded at 6.7%. Notably, INH showed the highest resistance, followed by streptomycin and rifampicin [12]. These findings support the recommendation to use a combination of four anti-tuberculous agents as empiric therapy to effectively address drug resistance in TB.

Oman has seen a slight rise in TB incidence, increasing from 8 to 11 cases per 100,000 population. Although this change is modest, it deserves attention and further investigation. Potential reasons for this increase may include enhanced detection methods, shifts in population demographics, or new challenges in TB control efforts. In addition, Oman has a significant expatriate population, many of whom come from countries with higher TB burdens such as India, Bangladesh, and Pakistan. Qatar, on the other hand, shows a significantly high TB incidence rate of 35 cases per 100,000 population. This elevated rate is concerning and may be linked to several factors, including a large migrant workforce from countries with high TB burden, difficulties in screening and treatment programs, or potentially improved detection methods that are identifying more cases. Oman has introduced screening LTBI at arrival screening for all expatriates with interferon-γ release assay (IGRA) and chest X-ray. It is crucial to implement targeted interventions and conduct thorough investigations to address the underlying causes of Qatar’s high TB rates. Enhanced diagnostic capabilities are crucial for addressing TB effectively. A study on culture-positive pulmonary TB offers valuable insights into the clinical presentation of pulmonary TB in Saudi Arabia [13]. Understanding these manifestations is vital for health care providers in the GCC because it facilitates early clinical suspicion and detection and improves treatment outcomes.

Mortality rates present a mixed picture in the GCC countries. SA has made significant milestone, reducing TB-related death by 32%. According to the Global Burden Diseases, Saudi Arabia had ≥−10% and <0% change in all forms of TB death from 2015 to 2020 [9]. In contrast, Qatar, Kuwait, and the UAE have seen increases in mortality rates (14%, 39%, and 22%, respectively). Treatment success rates vary widely from 100% in Qatar to 90% in Oman and 87% in Saudi Arabia. However, Bahrain (77%) and, particularly, Kuwait (44%) show opportunity for improvement in their treatment programs. This is in line with the global burden of diseases that showed only 17 countries achieved the End TB Strategy mortality milestone in 2020. Contributing factors for such suboptimal outcome include delays in detection and prompt treatment, suboptimal treatment adherence, unidentified drug resistance, and inadequate prevention of advanced HIV disease [9].

In controlling MDR-TB, Oman leads with a 78% reduction in MDR/RR-TB cases, followed by Bahrain (50%) and Qatar (40%), reflecting a significant reduction. The WHO had approved consolidated MDR-TB treatment guidelines focusing on effective and shorter duration anti-TB regimens that would play a role in reducing MDR-TB deaths [9].

Disparities in TB success within GCC countries are influenced by population and migration demographics, health care infrastructure including laboratory capacity, TB program implementation, socioeconomic factors, and cultural attitudes. In addition, there is potentially a variation in access to health care among vulnerable populations, such as migrant populations and PLHIV [4].

The GCC countries have a large expatriate workforce that comes originally from high–TB endemic areas [4]. Even with the advanced health care systems in the GCC countries, gaps in TB management and challenges may exist in these vulnerable populations. In Oman, for example, during the past 5 years, the average annual number of active TB cases was 356, with 71.4% of these new cases among migrants [14]; 65% had pulmonary TB and 80% had MDR-TB. This potentially affects treatment success rate and outcomes in the countries within the region.

The TB strain identified by whole genomic sequencing in Saudi Arabia and Oman have been linked to the non-nationals staying, mostly Delhi/CAS, Beijing, and EAI strains, that are mostly seen in the Indian subcontinent and Southeast Asian countries. This considerable genetic variability in the region is a challenge in TB control because these strains tend to display different responses to the treatment and drug resistance [10].

The prevalence of HIV-TB co-infection remains low in GCC countries compared with other regions. However, the rising number of new HIV cases may negatively impact TB treatment success rates. HIV-TB co-infection rates differ within the region, being highest in the UAE (5.4%) and Bahrain (5.1%), indicating a need for integrated HIV and TB services in these countries. Furthermore, the lower rates in Qatar (0.6%) and Kuwait (0.5%) may suggest more effective TB management programs in the HIV-TB co-infected population.

Using and implementing integrated approaches for HIV-TB, GCC countries can improve early detection, expedite treatment initiation, and enhance treatment adherence for TB and HIV. This not only benefits individual patients but also strengthens public health efforts to reduce transmission and achieve WHO elimination goals. Such integrated services can be effectively achieved through facility-based “one-stop” models, routine cross-screening, decentralization to community levels, robust data systems, and strong intersectoral collaboration. The NTP calls for dual screening for TB and HIV and is part of Saudi Arabia’s NTP [15]. This integration had been found to be useful in other countries as well [16].

Migrant workers face several challenges in accessing health care for TB, including limited access to health care services due to legal and financial barriers, fear of deportation or job loss after TB diagnosis, and language and cultural barriers that hinder effective communication with health care systems. In addition, poor living conditions, such as overcrowded accommodations, could exacerbate the risk of TB transmission and complicate timely detection and treatment. In a recent study on the etiologies of fever of unknown origin in the low- to middle-income countries, TB remains one of the most significant causes [17]. Early clinical suspicious and enhanced diagnostic capabilities are essential for prompt diagnosis of TB cases, which aligns with the overarching goals of the WHO End TB Strategy.

To address these challenges, GCC countries have implemented several migrant-friendly TB services. These include but not limited to the following: establishment of outreach health care programs, providing TB treatment free of charge to migrants, and using multilingual awareness campaigns and educational materials to bridge communication gaps. The introduction of directly observed therapy (DOT) in some countries has significantly improved treatment adherence among migrant populations. Moreover, most GCC countries conduct regular national campaigns aimed at raising awareness about TB and reducing stigma. These efforts collectively contribute to improving TB prevention, diagnosis, and treatment outcomes among vulnerable migrant populations.

Similarly, PLHIV have several challenges, such as stigma and discrimination, delayed diagnosis and treatment, managing the drug-drug interactions between antiretroviral therapy and TB medications, and high rates of drug resistance. To address these challenges, the GCC has implemented several supportive services. These services include integrated TB-HIV care where TB and HIV services are combined/co-located to ease the diagnosis and treatment, annual screening programs for LTBI among PLHIV and vice versa, and treatment of latent TB in PLHIV to reduce the risk of TB reactivation. Addressing these issues through targeted policies—such as improving access for migrant populations, strengthening NTPs, and reducing HIV stigma—can enhance treatment outcomes across the region.

The overall prevalence of LTBI in the MENA region was found to be 41.78% [6]. Although the GCC countries are classified as having a low incidence for TB [1,3,4], the LTBI incidence rate is 25-35% [18]. Countries in the region are making significant efforts to decrease TB rates even further by screening and treating LTBI. Strategies have been implemented to screen high-risk groups, including expatriates, health care workers, prisoners, and PLHIV. However, a significant number of LTBI cases are found among the foreign-born workforce population. Using different screening methods (Mantoux test, interferon-γ release assay, chest X-ray) before arrival, on arrival, and subsequent follow-up. Furthermore, treating LTBI is vital for elimination of TB, yet this population encounters substantial barriers, including limited access to universal health coverage, mostly among illegal migrants, issues of financial hardship, and fear of discrimination, which further impede their access to health care.

They are several other barriers in managing LTBI. The lengthy duration of therapy with the traditional treatment with INH often has low completion rates, necessitating the adoption of shorter treatment regimens. A 12-week regimen of weekly rifapentine and isoniazid has shown promise in improving patient adherence and preventing the progression to active TB [19] but cost and availability are potential hinders. Similarly, incorporating newer diagnostic tools, such as interferon-γ release assays, can enhance the accuracy of LTBI diagnoses, particularly, among individuals who have been vaccinated with Bacillus Calmette-Guerin; nevertheless, the cost is a challenge. Moreover, the rise of MDR/RR-TB poses a growing threat to effective TB management and could lead to an increase in the number of LTBI cases caused by MDR strains. Reports indicate that although MDR-TB is present among nationals in the GCC, the prevalence is significantly higher among the foreign-born workforce population [4,20,21].

To combat these challenges, it is essential to prioritize free preventive programs, including screening and treatment of LTBI in this population and ensure access to care across the GCC. Addressing MDR-TB requires a multifaceted strategy that include implementing effective TB infection control measures and promptly enrolling diagnosed patients in treatment to mitigate transmission risks. Considering drug susceptibility test results for *Mycobacterium tuberculosis* isolates from source cases is critical in selecting appropriate drugs for preventive therapy regimens. Enhancing surveillance to detect rifampicin sensitivity in all cases is also crucial.

To achieve the set goal of the WHO End TB Strategy of TB elimination by 2035, the GCC countries are required to adopt several recommendations that include strengthening TB surveillance systems, establishing partnerships and collaboration with international organizations such as WHO, engaging the non-governmental organizations and community-based organizations to deliver the culturally sensitive TB services, and investing in research and innovation. It is also advisable to reinforce the policies that protect the rights of migrants and PLHIV, ensuring their access to health care and prevention programs.

Policymakers should consider implementing integrated multidisciplinary programs that address LTBI and TB elimination efforts, with a focus on improving diagnostic capabilities. Expanding services through public-private partnerships can further strengthen TB elimination initiatives and alleviate financial pressures on governments. This activity may include DOTS strategy. However, one study from Saudi Arabia emphasizes the low rate of non-adherence to anti-tuberculous therapy under DOTS strategy. The findings underscore the necessity for robust adherence strategies to ensure treatment success and reduce the incidence of drug-resistant TB, which poses a significant challenge in the region [22].

Furthermore, the Gulf Health Council proposes several strategies that all GCC countries should adopt. These include strengthening the NTPs, managing LTBI treatment among foreign-born populations, and developing initiatives for vulnerable groups such as PLHIV, solid organ transplant recipients, and patients with diabetes mellitus. Establishing a robust monitoring and evaluation system for TB is essential, along with enhancing LTBI surveillance and prompt infection prevention measures. These actions are vital in preventing active TB and achieving the WHO End TB Strategy targets for 2025 and the United Nations Sustainable Development Goals for 2023 in the MENA region [6]. Furthermore, addressing MDR-TB effectively requires sustained political commitment, strong leadership, expanding partnerships, adequate funding for care and research, and ensuring the availability of essential drugs for shorter treatment regimens, such as bedaquiline and delamanid, through the Gulf Joint Procurement Program.

Among the review’s limitations are the absence of data on disease broken down by nationality and the fact that some of the countries’ statistics only apply to citizens of that country and excluded non-citizens.

## Conclusion

The GCC countries have made significant progress in improving diagnostic capabilities, screening contacts, and providing preventive therapy. However, their advancements toward the 2025 goals of the End TB Strategy vary widely, revealing successes and ongoing challenges in TB control. This diversity in outcomes highlights the need for enhanced regional collaboration.

Countries such as the UAE and Kuwait, which have implemented effective strategies, can share valuable insights and practices with their neighbors. Conversely, countries that are falling behind in certain areas could greatly benefit from adopting proven strategies used by their regional counterparts. Given the substantial migrant workforce in GCC countries, there is a pressing need for robust screening and treatment programs tailored to this demographic. Such initiatives are crucial for addressing the unique health challenges faced by this vulnerable population. The large migrant workforce, high prevalence of diabetes, and significant numbers of people living in congregate settings all contribute to increased TB vulnerability. Accurate surveillance systems remain essential for building on existing progress. Key interventions, such as reducing TB incidence, improving treatment success rates, managing drug-resistant TB, and addressing TB-HIV co-infection, are critical for GCC countries to collectively achieve the 2025 End TB Strategy goals. This collaborative approach, combined with targeted efforts in key areas, will be vital in the region’s ongoing fight against TB and its alignment with global TB elimination targets, including prospective future research common directions and the implications for policy.

## Declarations of competing interest

The authors have no competing interests to declare.

## References

[1] World Health Organization (2024).

[2] World Health Organization (2025).

[3] Al Awaidy S.T. (2018). Tuberculosis elimination in Oman: winning the war on the disease. ERJ Open Res.

[4] Al Awaidy S.T., Khamis F. (2018). Tuberculosis in Gulf Health Council Member States: opportunities and Challenges Towards TB elimination. Oman Med J.

[5] Awaidy S.A., Ghazy R.M., Mahomed O. (2023). Progress of the Gulf Cooperation Council (GCC) countries towards achieving the 95–95-95 UNAIDS targets: a review. J Epidemiol Glob Health.

[6] Barry M. (2021). Prevalence of latent tuberculosis infection in the Middle East and North Africa: a systematic review. Pulm Med.

[7] World Health Organization (2015). The end TB strategy. https://www.who.int/tb/strategy/end-tb/en/.

[8] World Health Organization (2021). Global tuberculosis report 2021. https://www.who.int/teams/global-tuberculosis-programme/tb-reports/global-tuberculosis-report-2021.

[9] GBD 2021 Tuberculosis Collaborators (2024). Global, regional, and national age-specific progress towards the 2020 milestones of the WHO End TB Strategy: a systematic analysis for the Global Burden of Disease Study 2021. Lancet Infect Dis.

[10] Al Bati N.A. (2024). Impact of migrant populations on tuberculosis rates in Saudi Arabia: assessing how migration patterns affect TB incidence and control measures: a narrative review. J Tuberc Res.

[11] Alawi M.M., Alserehi H.A., Ali A.O., Albalawi A.M., Alanizi M.K., Nabet F.M. (2024). Epidemiology of tuberculosis in Saudi Arabia following the implementation of end tuberculosis strategy: analysis of the surveillance data 2015–2019. Saudi Med J.

[12] Al-Tawfiq J.A., Hinedi K., Memish ZA. (2015). Systematic review of the prevalence of Mycobacterium tuberculosis resistance in Saudi Arabia. J Chemother.

[13] Al-Tawfiq J.A., Saadeh BM. (2009). Radiographic manifestations of culture-positive pulmonary tuberculosis: cavitary or non-cavitary?. Int J Tuberc Lung Dis.

[14] Alyaquobi F.M., Alhakamani F., Alsabari M., Althuhli K., Al AlMahroqi M.A., Al-Jardani A. (2025). A step forward in tuberculosis elimination: implementing migrant latent tuberculosis screening and treatment in Oman. IJID Reg.

[15] Ministry of Health (2021). National tuberculosis program manual. https://www.moh.gov.sa/Documents/National-TB-program.pdf.

[16] Hermans S.M., Castelnuovo B., Katabira C., Mbidde P., Lange J.M., Hoepelman A.I. (2012). Integration of HIV and TB services results in improved TB treatment outcomes and earlier prioritized ART initiation in a large urban HIV clinic in Uganda. J Acquir Immune Defic Syndr.

[17] Erdem H., Al-Tawfiq J.A., Abid M., Yahia W.B., Akafity G., Ramadan M.E. (2024). Infectious causes of fever of unknown origin in developing countries: an international ID-IRI study. J Intensive Med.

[18] Ding C., Hu M., Guo W., Hu W., Li X., Wang S. (2022). Prevalence trends of latent tuberculosis infection at the global, regional, and country levels from 1990–2019. Int J Infect Dis.

[19] Alvarez G.G., Sullivan K., Pease C., Van Dyk D., Mallick R., Taljaard M. (2022). Effect of implementation of a 12-dose once-weekly treatment (3HP) in addition to standard regimens to prevent TB on completion rates: interrupted time series design. Int J Infect Dis.

[20] Singh J., Al-Abri S., Petersen E., Al Yaqoubi F., Al Rahbi K., Al Balushi L. (2022). Importance of tuberculosis screening of resident visa applicants in low TB incidence settings: experience from Oman. J Epidemiol Glob Health.

[21] Ali Chaudhry L., Rambhala N., Al-Shammri A.S., Al-Tawfiq J.A. (2012). Patterns of antituberculous drug resistance in Eastern Saudi Arabia: a 7-year surveillance study from 1/2003 to 6/2010. J Epidemiol Glob Health.

[22] Chaudhry L.A., Al-Tawfiq J., Ba-Essa E., Robert AA. (2015). Low rate of non-compliance to antituberculous therapy under the banner of directly observed treatment short course (DOTS) strategy and well organized retrieval system: a call for implementation of this strategy at all DOTS centers in Saudi Arabia. Pan Afr Med J.

